# Application of anodized titanium for enhanced recruitment of endothelial progenitor cells

**DOI:** 10.1186/1556-276X-7-298

**Published:** 2012-06-07

**Authors:** Iman Moradi, Mohaddeseh Behjati, Mohammad Kazemi

**Affiliations:** 1 Nanotechnology Consultancy & Development Center (NCDC), Via Svizzera 16, Padova, 35127, Italy; 2 Department of Cardiology, Isfahan University of Medical Sciences, Isfahan, 8174673461, Iran; 3 Isfahan University of Medical Sciences, Isfahan, 8174673461, Iran

**Keywords:** endothelial progenitor cell, cell attachment, cell proliferation, anodized titanium

## Abstract

**Objectives:**

To study the efficacy of an effective anodized titanium surface with enhanced attachment of endothelial progenitor cell (EPC).

**Background:**

In-stent restenosis is a major obstacle for vascular patency after catheter-based intravascular interventions. Recently, stents that capture EPCs have been paid attention in order to make a functional endothelialized layer at the site of stent-induced endothelial denudation. Anodized titanium has been shown to enhance stem cell attachment. Anodization is a quick and inexpensive method, which can provide suitable stent surface.

**Methods:**

Surface topography was examined by high-resolution scanning electron microscopy (SEM). Substrates were co-cultured with EPCs at second passage in 24-well culture plates. Evaluation of cell growth, proliferation, viability, surface cytotoxicity and cell adhesion was performed using 3-(4,5-dimethylthiazol-2-yl)-2,5-diphenyltetrazolium bromide (MTT) test and 4,6-diamidino-2-phenylindole dihydrochloride staining. For platelet attachment, platelets added to substrates were evaluated under SEM.

**Results:**

The average MTT values for tissue culture polystyrene plate, unanodized and anodized titanium with nanostructure were equal to 0.49, 0.16 and 0.72, respectively (*P* < 0.05). The surface had no cytotoxic effects on cells. The average cell attachment results showed that 9,955 ± 461.18, 3,300 ± 197.98 and 11,359 ± 458.10 EPCs were attached per well of tissue culture polystyrene plate, unanodized and anodized titanium surfaces, respectively (*P* < 0.05).

**Conclusions:**

Anodized titanium surfaces can be potentially applied for devices that need enhanced recruitment of EPCs. This unique property makes these anodized surfaces good and cheap candidates for designing cardiovascular medical devices as endovascular stents.

## Background

There is a great competition to find ideal scaffolds for tissue-engineering approaches. Ideal scaffold should meet the criteria as being cytocompatible, matched with surrounding tissues and able to provide chemical stability or degradability, affording mechanical strength and allowing cell adhesion and proliferation. Biomaterial scaffolds are designed and tested for one particular or various cell types. Currently, there is a great enthusiasm toward attracting desired stem cells to the surface of metallic devices as part of tissue-engineering applications especially in the field of regenerative medicine. Medical devices with cardiovascular applications are intended generally to attract regenerative endothelial progenitor cells (EPCs) to the surface of metal. Homed and attracted EPCs augment surface endothelialization, which consequently leads to lesser thrombotic events triggered by the coarse metallic surface of a device [1].

Titanium, a smart biomaterial with specific characteristics, has been used frequently in both basic research and medical devices. Titanium is among the metals with wide application in the field of cardiovascular devices due to its biocompatibility and shape elastic memory properties [2]. This wide application is attributed to its excellent corrosion resistance and suitable mechanical properties [3]. Titanium showed good properties to be a suitable scaffold for tissue-engineering applications. Drawbacks related to the limited functional lifetimes of titanium-based devices have been proposed to be solved using nanostructured materials [4]. Indeed, some surface treatment procedures might enhance adhesion of desired cells to this metal. The search to find the most effective treatment of titanium is still continuing.

Among surface modification processes, anodization (anodic oxidation) provides nanotextured features on metal oxides [5]. This process leads to the formation of protective TiO_2_ nanostructures on the surface, which provides a natural environment for cell growth [5]. The increased thickness and stability of this formed oxide layer on titanium protect it from uncontrolled oxidation, chemical reactions and corrosion [6]. Topography of metal surface is easily changed by anodization [6]. In this way, bioactivity and biocompatibility of the underlying metal is enhanced. The absence of elicitation of inflammatory reactions of anodized treaded surfaces is also demonstrated previously [5]. Despite several advantages of anodized surfaces, this technique does not meet cost-benefit ratio measures due to the need for expensive platinum to serve as cathode within anodization process in nanoscales [7]. Thus, in this article, we present an effective anodized titanium surface that provides a safe and nontoxic surface with enhanced EPC attachment and proliferation while the technique is cheap enough because of using steel as the cathode. To the best of our knowledge, this is the first time that a modified anodized titanium surface has been used for this purpose.

## Methods

### Sample preparation

A sheet of commercially pure titanium grade 1 ASTM B 265 (William Gregory Ltd., UK) with a thickness of 1 mm was cut into 8 × 5 cm pieces using an automatic metal cutting machine. The titanium pieces were cleaned with liquid soap and 70% ethanol for 10 min in an ultrasonic bath. The substances were dried in an oven at about 658°C for 30 min in order to prepare them for anodization. After anodization, substrates were ultrasonically washed in an ultrasonic bath with acetone for 20 min and 70% ethanol for 20 min [1]. To remove naturally formed oxide layers, substrates were immersed in an acid mixture containing 2 ml 48% HF, 3 ml 70% HNO_3_ and 100 ml deionized (DI) water for 5 min prior to anodization. Some of the acid-polished substrates were treated immediately by anodization. Titanium substrates were used as an anode in anodization process, while a stainless steel sheet served as a cathode. What makes this method cheaper is the application of stainless steel instead of platinum as a cathode pole, which makes the technique more commercial (Patent no. V12011A000166). Anode and cathode poles were connected by copper wires and linked to positive and negative ports of a 30 V/3 A power supply, respectively. While processing, anode and cathode poles, kept in parallel with a separation distance of about 2 cm, were submerged into an electrolyte solution of diluted HF (1.5 wt.%) in a Teflon beaker. A constant potential between the anode and cathode poles at 20 V was applied. After accomplishing the anodization process within 20 min, the substrates were rinsed thoroughly with DI and dried in an oven at about 658°C for 30 min. Anodized titanium nanostructure was then cut into small pieces of 1 × 1 cm^2^ with an automatic cutting machine. Sheets were then cleaned serially with tetrachloroethylene, 20 min in acetone and 20 min in 70% ethanol in ultrasonic bath. Treatment with UV for 30 min was applied for sterilization of the anodized titanium nanostructure pieces. A piece of unanodized 1 × 1 cm^2^ titanium sample was used as a control after cleaning through the above-mentioned serial procedure. Indeed, one tissue culture polystyrene plate also served as a control surface [7].

### Isolation and characterization of EPCs

Thirty milliliters of heparinized blood from human umbilical cord was used to isolate EPCs. Briefly, blood was diluted 1:1 with phosphate buffered saline (PBS) and overlayed on Lymphoprep (Lymphoprep™, 1114544, Gentaur, Kampenhaut, Belgium). Cells were centrifuged at 600 g for 20 min. The resulting mononuclear cells (MNCs) were collected and washed three times with PBS. One million isolated MNCs were resuspended in endothelial basal medium-2 (EBM-2) supplemented with EGM-2 bullet kit (CC-3162, Lonza Milano, Treviglio, Italy), plated on a T-25 culture flask coated with 10 μg/ml human fibronectin (F2006, Sigma-Aldrich, St. Louis, MO, USA). Non-adherent cells were removed after culture for 3 days, and fresh medium was added. The medium was replaced every 3 days during the entire culture period. Attached EPC appeared elongated with a spindle shape. Cells were phenotypically characterized using flow cytometry by co-expression of CD34, KDR and CD146 surface markers. The following antibodies have been used in order to characterize the EPCs: PE-conjugated anti-human CD34 antibody (SC-7324, SANTA CRUZ Biotecnology, Inc., Heidelberg, Germany), a PE-conjugated anti-human KDR antibody (FAB375P, R&D SYSTEMS, Minneapolis, MN, USA), an FITC-conjugated anti-human CD146 antibody (11-1469, eBioscience, San Diego, CA, USA) and a PE-CY5-conjugated anti-human CD45 (SC-18901, SANTA CRUZ Biotechnology) [8].

### Cell experiments

EPCs at the second passage were trypsinized by reaching 80% of their confluency. By 8 min centrifugation at 1,800 rpm, cell suspension was transferred into 5 ml of the culture media. Control and test substrates were inserted at the bottom of 24-well tissue culture plates. Plates were incubated at 37°C and 5% CO_2_. The cells were used for evaluation of cell attachment, cell growth and proliferation. For each evaluation, all experiments were run in triplicate.

### Cell growth, proliferation, viability and surface cytotoxicity

One milliliter of the culture media containing 2 × 10^4^ cells was cultured on the substrate surface in 12-well plates for 48 h. The plates were incubated in standard cell culture conditions at 37°C temperature and 5% CO_2_ for 48 h. Culture media were removed from wells. Then, 900 μl of the culture media was added to the wells. Subsequently, 100 μl of 5 mg/ml 3-(4,5-dimethylthiazol-2-yl)-2,5-diphenyltetrazolium bromide (MTT) (final concentration, 0.5 mg/ml) was added to the wells, and the cells were incubated in CO_2_ incubators for 4 h. Culture media were removed, and 1 ml of acidic isopropanol was added to each well. After 10 min of incubation at 37°C, cell supernatant was transferred into the 1.5-ml tube, and cell suspension was made by centrifugation at 14,000 rpm for 2 min. Supernatant was used for analysis by ELISA reader in 570 and 630 nm (reference wave length) [9].

### Cell attachment

Cells (5 × 10^4^/well) were kept in an incubator in 12-well plates with substrates. After 4 h, culture media were removed and cells were washed twice with PBS. Then, cells were treated with 4% paraformaldehyde for 30 min at 4°C. After washing with PBS, cells were treated with 4,6-diamidino-2-phenylindole dihydrochloride (DAPI) in a dark room for 15 min. Then, cells were washed twice with PBS, and cells attached to the substrates were counted by fluorescence microscope. Untreated tissue culture polystyrene was used as a negative control.

### Platelet attachment

Platelet-rich plasma (PRP) was prepared by centrifugation of EDTA-anticoagulated blood at 1,000 rpm for 10 min. The upper layer PRP was transferred into the fresh clean tube, and platelets were counted using a hemocytometer. Platelets were diluted in PBS up to 200,000/μl. Then, 1 ml of PBS was added into the wells. After 1 hr of incubation at 37°C, cells were gently washed with 1 ml of PBS. Cells were fixed in 2.5% gluthardehyde for 1 h at 37°C [9]. Thereafter, dehydration step was performed using 1 ml of accelerated ethanol concentration (15 min for each concentration) as follows: 50, 60, 70, 80, 90 and 100 (two times). Finally, substrates containing cells were dried at room temperature and studied using scanning electron microscopy. Untreated substrate was used as negative control. Counting cells attached to the substrate is going through the following formula: cell counts attached on substrates/cell counts on control wells.

### Electron microscopic evaluation

Cells (5 × 10^4^/well) were incubated in a CO_2_ incubator in 12-well plates with substrates for 4 h. Then, culture media were removed and washed twice with 1 ml of PBS. Cells were incubated in room temperature after the addition of 1 ml of 2.5% gluthardehyde. After 2 h, gluthardehyde was removed and the substrates were washed with PBS twice. Dehydration step was performed using 1 ml of accelerated ethanol concentration (15 min for each concentration) as follows: 50, 60, 70, 80, 90 and 100 (two times). Finally, substrates containing cells were dried at room temperature and studied using scanning electron microscopy. Untreated substrate was used as negative control.

### Statistical analysis

Numerical data were analyzed through analysis of variance via SPSS software. *P* values less than 0.05 were considered statistically significant.

## Results

**Figure 1:**
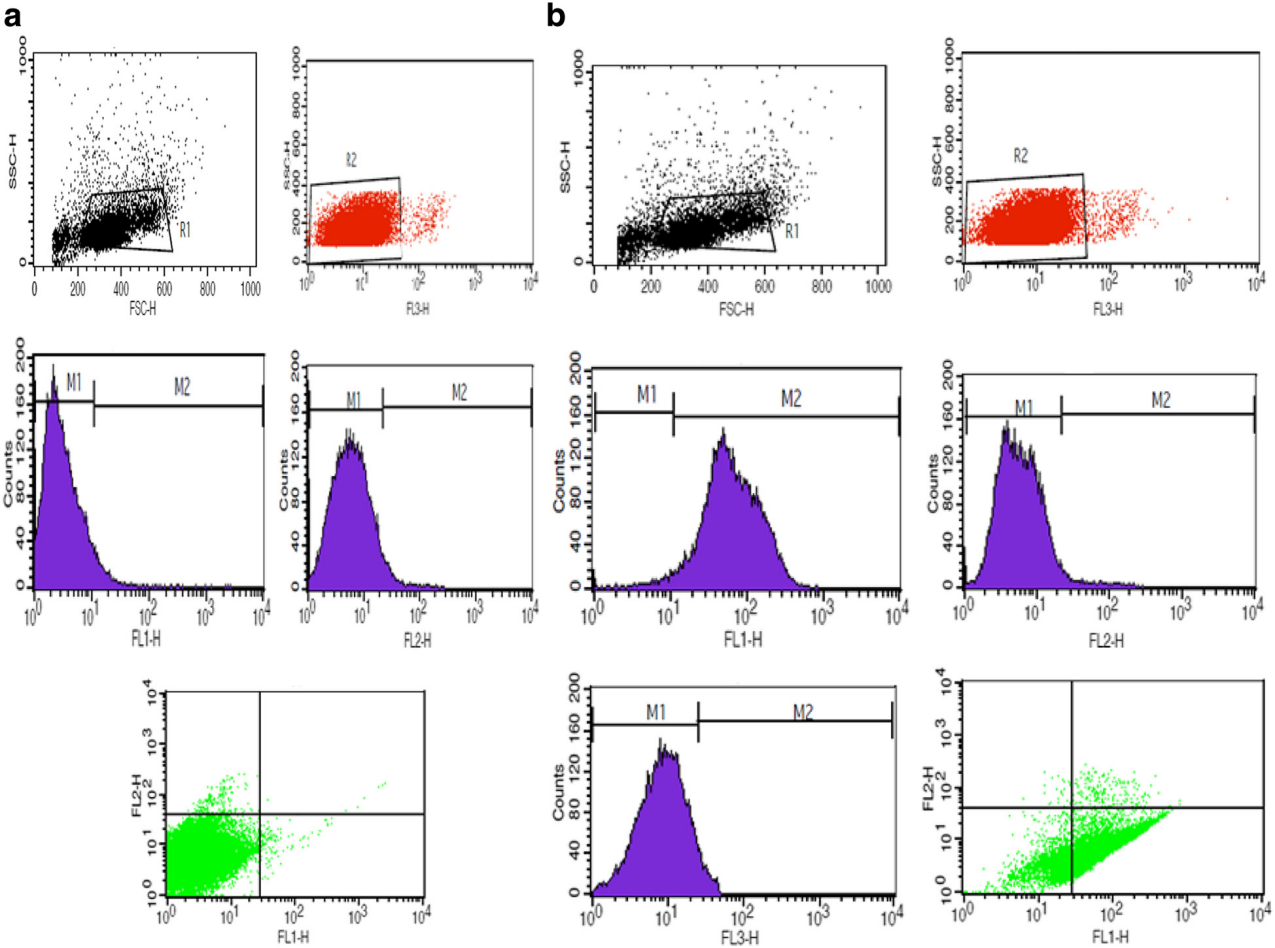
**Characterization of isolated endothelial progenitor cells.** Flow cytometric analysis of surface markers of EPCs using FITC-conjugated anti-CD146, PE-conjugated anti-CD34 and PE-Cy5-conjugated anti-CD45 monoclonal antibodies demonstrated that 78% **(A)** and 80% **(B)** of the cells were triple positive for the markers. FLI, FL2 and FL3 represent CD146, KDR and CD45 expressive cells, respectively. The percentage of expressing cells was calculated using the cell quest program.

**Figure 2:**
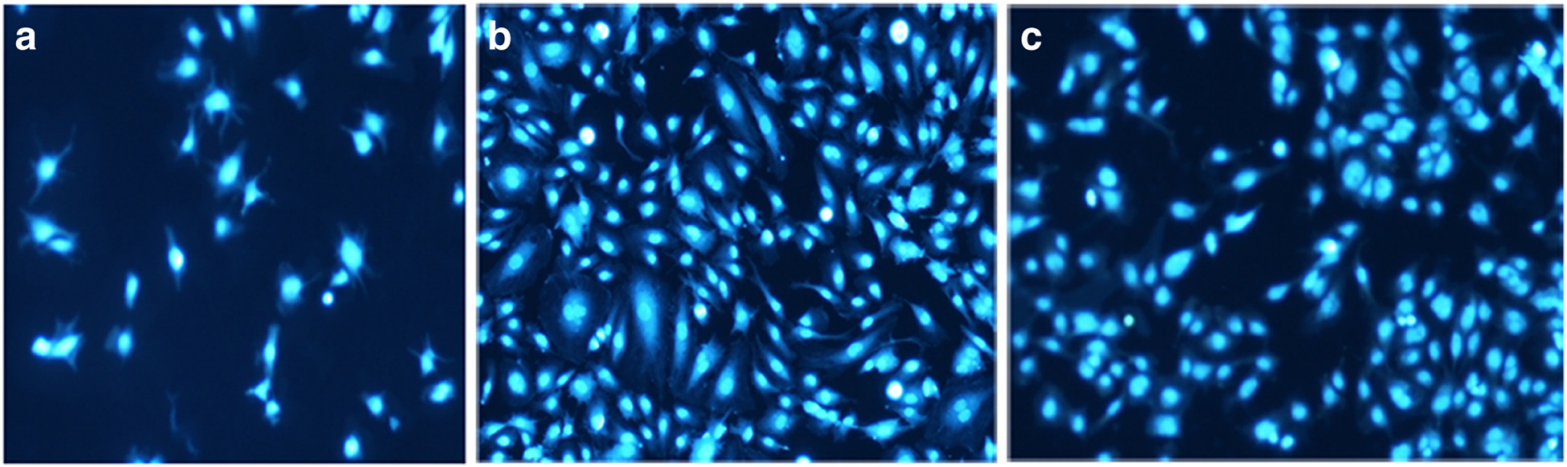
**EPCs’ adhesive behavior in co-cultures with unanodized, anodized titanium surfaces and tissue culture polystyrene plates.** Fluorescent images of increased EPC adhesion on TCPS (upper panel), unanodized titanium (middle panel) and anodized titanium with nanostructures (lower panel). Cell nuclei are stained with DAPI.

Flow cytometric analysis showed that most of the cells (79%) were triple positive for CD34, CD146 and CD45 surface markers, which are characteristic for EPC. Figure 1 demonstrates flow cytometry characteristics of isolated EPCs. From triplicate tests performed for cell proliferation and viability, the average MTT values for tissue culture polystyrene (TCPS), unanodized and anodized titanium surfaces were equal to 0.49, 0.16 and 0.72, respectively. The proportion of MTT for cells cultured on anodized titanium surface was 1.46-fold of the TCPS, which was significantly higher than unanodized titanium surface with MTT value equal to 0.33-fold of the TCPS (*P* < 0.05) (anodized titanium/control surface). This reflects the higher cell growth, proliferation and viability on this anodized titanium surface compared with the control samples (*P* < 0.05). The average cell attachment results obtained from triplicate DAPI staining showed that 9,955 ± 461.18, 3,300 ± 197.98 and 11,359 ± 458.099 EPCs were attached per square centimeter of TCPS, unanodized and anodized titanium surfaces, respectively. Cell adhesion to anodized titanium was equal to 1.14-fold of the TCPS, which was significantly higher than unanodized titanium with 0.33-fold of the TCPS (*P* < 0.05). Figure 2 represents DAPI-stained EPCs on TCPS, unanodized and anodized titanium. The surface had no cytotoxic effects on the cells.

**Figure 3:**
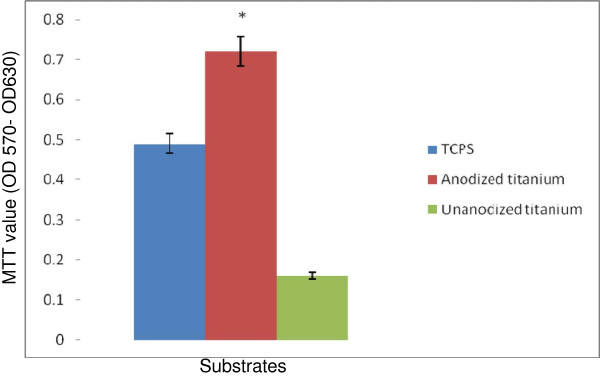
**Cell growth, proliferation, viability and surface cytotoxicity.** Cell survival and proliferation on TCPS, unanodized titanium and anodized titanium with nanostructures. Cell survival and proliferation were evaluated using MTT test. MTT measure in *Y* axis means values in relative quantity. Asterisk denotes *P* < 0.05. Data are mean ± SEM, *n* = 3.

**Figure 4:**
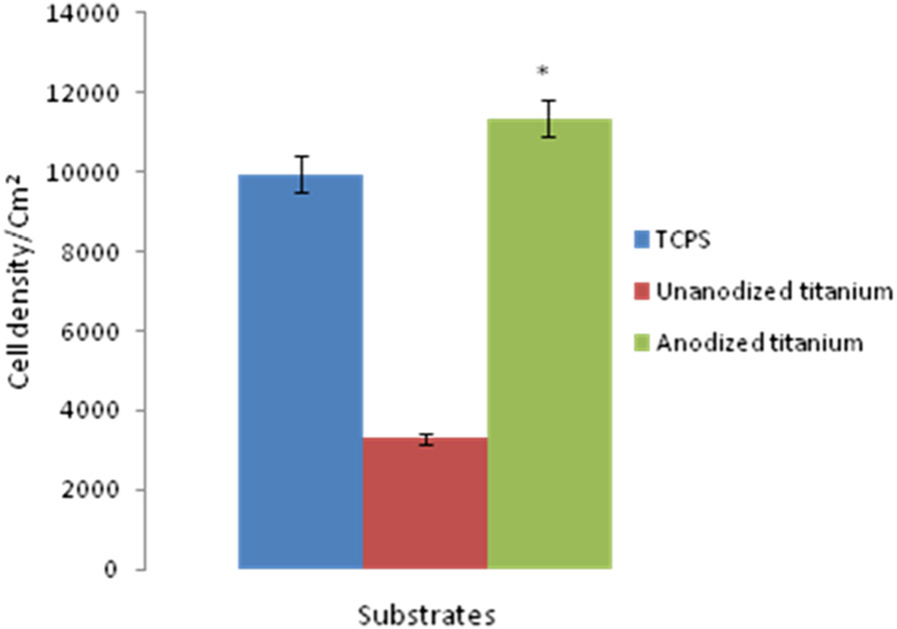
**Cell attachment on applied surfaces.** The density of attached cells to TCPS (*n* = 14), unanodized titanium (*n* = 3) and anodized titanium with nanostructures (*n* = 18). Evaluation of cell adhesion was conducted by counting stained nuclei of attached cell per square centimeter of the substrate surface. Asterisk denotes *P* < 0.05. Data are mean ± SEM.

**Figure 5:**
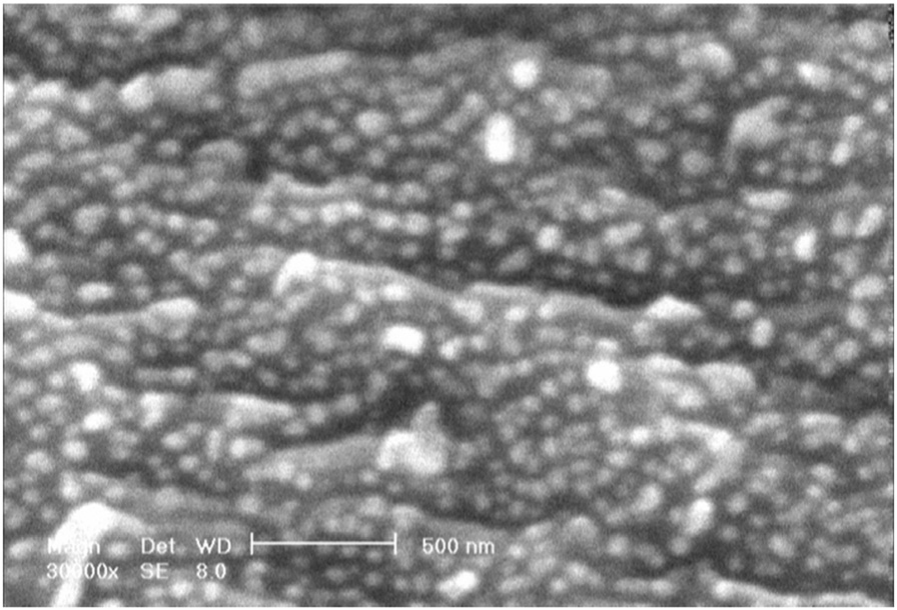
**Nanostructured anodized titanium at high magnification.** High-magnification SEM picture of anodized titanium surface. Photo was taken at high magnification (×30,000). Bar 500 nm.

**Figure 6:**
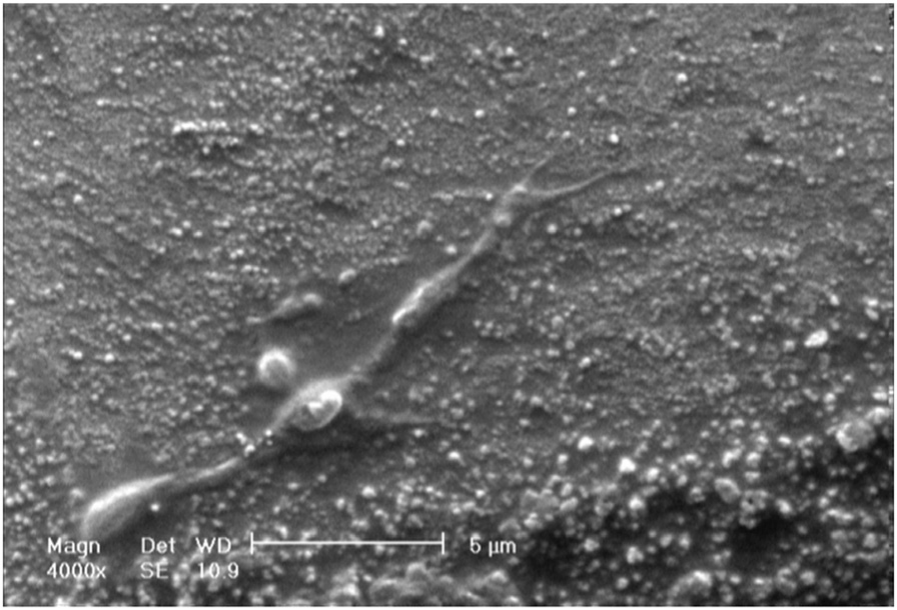
**Flattened endothelial progenitor cells on the surface of nanostructured anodized titanium.** SEM picture of attached and flattened EPC on anodized titanium surfaces after 48 h (×4,000). Bar 600 nm.

**Figure 7:**
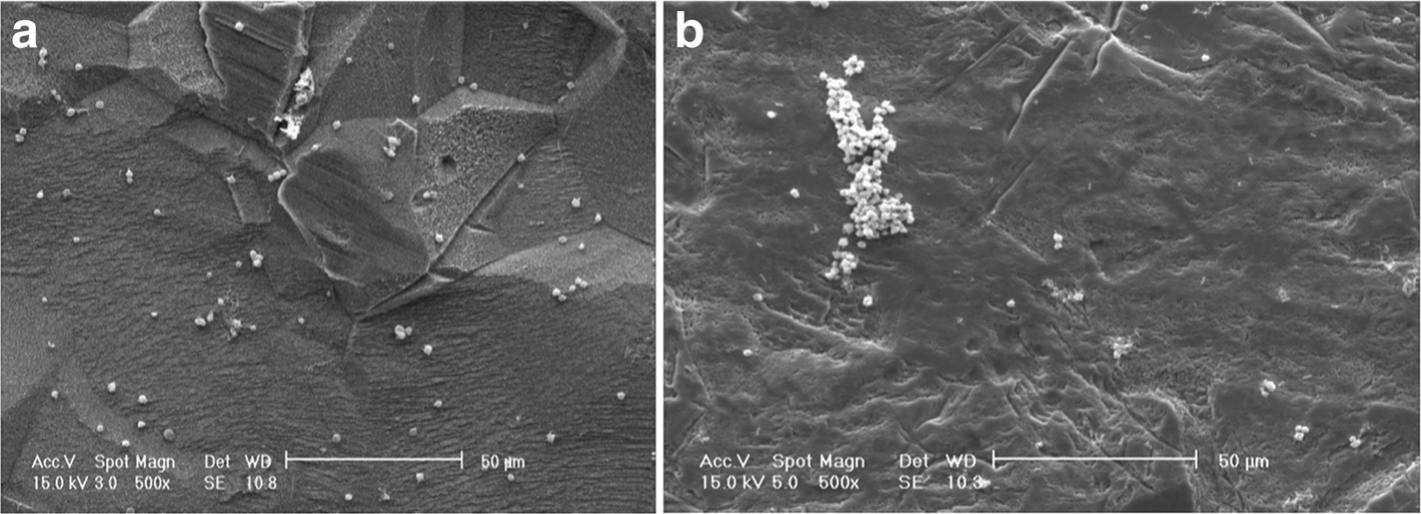
**Platelet attachment on the surface of anodized and unanodized titanium.** Platelet attachment on the surface of anodized titanium surface (panel **A**) after 4 h compared with unanodized titanium (panel **B**) (×500).

Data in Figures 3 and 4 represent the measures of cell survival, proliferation and adhesion on different investigated surfaces in this experiment. High-magnification (×30,000) scanning electron microscopy (SEM) picture of an anodized titanium surface is shown in Figure 5. Figure 6 demonstrates an SEM picture of an attached and flattened EPC on anodized titanium surface compared with unanodized titanium surface (×4,000). Data in Figure 7 demonstrate the lower platelet attachment on the surface of anodized vs. untreated titanium surface (×500).

## Discussion

Our data demonstrate promise for application of anodized titanium in order to enhance the attachment of EPCs. By now, several treated surfaces have been examined for this purpose, but these surfaces are dramatically inexpensive. Changes in surface topography after titanium anodization might influence EPC adhesion. Presence of the formed protective TiO_2_ nanostructures on the surface of the substrate contributes to enhanced EPC attachment and proliferation through mimicking natural nanoscale features for these cells. The unique nanostructure in this treated substance, as demonstrated in the SEM photograph, provides more surface area and reactive sites for enhanced cell adhesion. The results of this investigation suggest that anodized nanostructure surface topography resulting from titanium leads to greater EPC adhesion and proliferation. Since nanostructure designed in this substrate allows sustained drug release, this surface can be used for indications in which enhanced EPC adhesions are desired with simultaneous drug delivery.

This surface, designed using an inexpensive technology, allows its appropriate application for medical devices intended for cardiovascular applications. Enhanced EPC adhesion on cardiovascular devices brings the opportunity of accelerated endothelialization, which consequently hinders thrombus formation [1]. One of the important prerequisites for a successful alloy used for cardiovascular medical devices, apart from attraction of EPCs, is deferring macrophages and inflammatory cells to reduce in-stent thrombosis and thrombosis. Anodized titanium surface, as a biocompatible and bioinert surface, is not associated with elicitation of untoward local and systemic inflammatory reactions [5]. Reported reduced macrophage density on anodized titanium surface by Rajyalakshmi et al. provides further evidence for its efficacy for cardiovascular devices [5]. Inflammatory reactions due to the interaction between medical device and the host tissue lead to long-term success failure. In this case, metal devices with impregnated polymeric material impose greater inflammatory burden [10]. As an example, enhanced inflammatory reactions, both locally and systemically, to the implanted stents lead to accelerated rate of stent thrombosis and restenosis [10]. This technique avoids application of non-erodible polymers for endovascular stents, and metal surface could be exposed directly to the blood flow. Parallel to more EPC adhesion, decreased platelet attachment to this surface makes this a desirable surface for endovascular applications.

Thus, our data are in favor of the application of anodized titanium surface created through this effective method for cardiovascular medical devices. This method is much cheaper through replacing platinum with stainless steel to serve as a cathode pole in the anodization process. This method avoids chemical modification of the surface, which might be harmful for cell health.

## Conclusions

Anodized nanostructured titanium surfaces can be potentially applied for enhanced recruitment of EPCs via formation of the protective TiO_2_ layer. This unique property makes these anodized surfaces good candidates for designing cardiovascular medical devices as endovascular stents.

## Abbreviations

DAPI, 4,6-diamidino-2-phenylindole dihydrochloride; DI, Deionized water; EBM-2, Endothelial basal medium-2; EDTA, Ethylene-amine tetra-acetic acid; EGM-2, Endothelial growth medium-2; ELISA, Enzyme-linked immunosorbent assay; EPC, Endothelial progenitor cell; MNCs, Mononuclear cells; MTT, 3-(4,5-dimethylthiazol-2-yl)-2,5-diphenyltetrazolium bromide; PBS, Phosphate buffered saline; PRP, Platelet-rich plasma; SEM, Scanning electron microscopy; TCPS, Tissue culture polystyrene; TiO2, Titanium dioxide.

## Competing interests

The authors declare that they have no competing interests.

## Authors’ contributions

IM carried out the nanostructure fabrication and participated in the design of the study. MB carried out the designation of the study and the preparation, isolation and characterization of EPCs. MK participated in the preparation, isolation and characterization of EPCs. All authors read and approved the final manuscript.
